# Knowing with Which Eye We See: Utrocular Discrimination and Eye-Specific Signals in Human Visual Cortex

**DOI:** 10.1371/journal.pone.0013775

**Published:** 2010-10-29

**Authors:** Dietrich Samuel Schwarzkopf, Andreas Schindler, Geraint Rees

**Affiliations:** 1 Institute of Cognitive Neuroscience, University College London, London, United Kingdom; 2 Wellcome Trust Centre for Neuroimaging, University College London, London, United Kingdom; 3 Werner Reichardt Centre for Integrative Neuroscience/Max Planck Institute for Biological Cybernetics, Tübingen, Germany; National Institute of Mental Health, United States of America

## Abstract

Neurophysiological and behavioral reports converge to suggest that monocular neurons in the primary visual cortex are biased toward low spatial frequencies, while binocular neurons favor high spatial frequencies. Here we tested this hypothesis with functional magnetic resonance imaging (fMRI). Human participants viewed flickering gratings at one of two spatial frequencies presented to either the left or the right eye, and judged which of the two eyes was being stimulated (utrocular discrimination). Using multivoxel pattern analysis we found that local spatial patterns of signals in primary visual cortex (V1) allowed successful decoding of the eye-of-origin. Decoding was above chance for low but not high spatial frequencies, confirming the presence of a bias reported by animal studies in human visual cortex. Behaviorally, we found that reliable judgment of the eye-of-origin did not depend on spatial frequency. We further analyzed the mean response in visual cortex to our stimuli and revealed a weak difference between left and right eye stimulation. Our results are thus consistent with the interpretation that participants use overall levels of neural activity in visual cortex, perhaps arising due to local luminance differences, to judge the eye-of-origin. Taken together, we show that it is possible to decode eye-specific voxel pattern information in visual cortex but, at least in healthy participants with normal binocular vision, these patterns are unrelated to awareness of which eye is being stimulated.

## Introduction

When only one of the eyes receives visual stimulation, normal human observers sometimes report a “feeling of strain” or a presence in this eye allowing them to guess with which eye they saw the stimulus [1]–[5]. This phenomenon is known as utrocular discrimination [1] and reveals the extent to which low-level monocular signals in the visual system can be used to guide accurate discrimination.

Anecdotal reports suggest that stereoblind observers (usually with a history of early onset strabismus) know, or can even control, with which eye they are seeing. A systematic study of the dependency of utrocular discrimination on the state of binocular vision found that normal observers were only able to report the eye-of-origin for low spatial frequency stimuli, but that performance became progressively worse when spatial frequency was increased until it reached chance levels [5], [4]. On the other hand, stereoblind observers with matched visual acuity exhibited good utrocular discrimination at all spatial frequencies tested. The authors reasoned that the stereoblind visual system, which contains mostly monocular neurons that respond to only one of the eyes [6], [7] can read out monocular signals across the range of spatial frequencies. Conversely, they interpreted the result for normal observers as evidence that in the normal visual system the monocularity of neurons is related to spatial frequency tuning.

Neurophysiological studies and anatomical experiments confirm that there may indeed be such a dependency. Early studies reported that in the primary visual cortex (area V1) of non-human primates and cats neuronal populations tuned to low spatial frequencies coincide with the location of cytochrome oxidase (CO) “blobs” [8]–[10]. Moreover, in many mammals, including humans, V1 is segregated into ocular dominance columns that contain predominantly monocular cells driven by only one of the eyes [11], [12]. Further research shows that CO blobs are usually found at the center of ocular dominance columns [8], [13], [14]. By extension this means that low spatial frequency neurons also cluster at the center of ocular dominance columns and should thus correspond to precisely the most monocular neuronal populations. One study used optical imaging of intrinsic signals to compare the preference maps for ocular dominance, orientation and spatial frequency [15], which showed that there is indeed a weak correspondence between low spatial frequency domains and ocular dominance columns.

**Figure 1:**
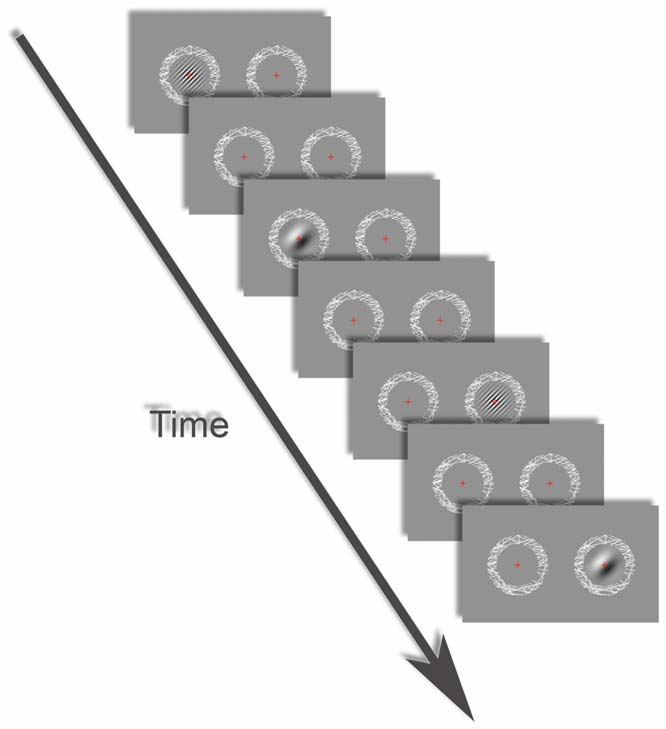
Illustration of the stimuli used in the experiment. Participants used free fusion to view the two rings comprising random line patterns on each side of the screen. On separate trials, we either stimulated the left or right eye, with a low or high spatial frequency grating presented inside the ring. The gratings flickered by reversing contrast polarity. In each fMRI run each stimulus condition was shown only once (as depicted here, presented in a pseudo-randomized order) and each trial lasted 19.2 s. In the behavioral experiments outside the scanner trials lasted 350 ms and 624 trials were presented per run. Stimulation trials were interleaved with fixation periods in which only the fusion rings and fixation crosses were presented.

Direct investigations of this relationship in the human visual system are still lacking. Here we employed high field high spatial resolution functional magnetic resonance imaging (fMRI) and behavioral measurements to address these questions. Specifically, we sought to examine whether monocular responses in human primary visual cortex were more selective for low rather than high spatial frequencies; and whether there was any systematic relationship between behavioral utrocular discrimination and the monocularity of neuronal populations in the early human visual cortex. We presented participants with small gratings of either low or high spatial frequency that were shown either to the left or right eye, whilst the other eye only saw a uniform grey (Figure 1). Brain activity was measured using Blood Oxygenation Level Dependent (BOLD) fMRI while participants indicated the eye to which they thought the grating had been presented. We employed multivoxel pattern analysis (MVPA: [16]–[18]) using a simple correlation algorithm to test for weak but consistent biases of individual voxels in local spatial patterns of activity evoked by the gratings. We hypothesized that decoding accuracy for discriminating voxel response patterns to left and right eye stimulation in V1 should be more reliable for low spatial frequency gratings. We further surmised that in accordance with previous studies [5], [4], behavioral accuracy for discriminating which eye saw the stimulus would be better for low than for high spatial frequencies.

## Methods

### Participants

All procedures were approved by the local ethics committee (Institute of Neurology and National Hospital Joint Ethics Committee at UCL) and participants were treated in accordance with the Declaration of Helsinki. Ten healthy participants (4 female, 2 left-handed, age: 20–35) with normal or corrected-to-normal vision gave written informed consent to participate in this experiment. They were naïve to the purpose of the experiment, except for one of the authors (DSS). Data from one participant was excluded from analyses because of excessive head motion and severe EPI distortions; another participant was excluded because of being unable to maintain stereo-fusion.

### Stimuli

Through a mirror attached to the headcoil, participants viewed flickering Gabor patches, i.e. a sinusoidal carrier grating convolved with a Gaussian aperture subtending approx. 3°- (standard deviation of Gaussian: 0.7°-) in which contrast-polarity reversed at 5 Hz with saw tooth wave modulation. Stimuli were presented at a resolution of 1024*768 on a uniform grey screen. Stimuli were presented at two spatial frequencies: a low frequency (0.5 cycles/°-) and a high frequency (3.6 cycles/°-). Moreover, stimuli could be presented either to the left or the right eye (Figure 1). This was achieved by means of free stereo-fusion and aided by a black foam board divider placed into the back of the bore in the space between the viewing mirror and the screen at the rear of the scanner. The divider was covered with black cloth to prevent reflections of the stimulus. The orientation of the carrier grating was always 45°- clockwise from vertical. Spatial phase of the Gabors was held constant which helped participants to maintain stereo-fusion. On each side of the divider, a ring comprising randomly oriented lines and a red fixation cross served as a fusion aid. The fusion ring subtended 7.7°-. The distance between the images of the ring (and stimulus) for the two eyes was adjusted prior to scanning for every participant to optimize comfort and good stereo-fusion. The luminance range of the display was not linearized (minimum: 0.4 cd/m^2^; maximum: 166 cd/m^2^). While this means that the mean luminance of the stimuli was slightly different from the background (36 cd/m^2^), crucially, mean luminance was matched between the two spatial frequencies (39 cd/m^2^). All stimuli were presented using the Cogent toolbox (http://www.vislab.ucl.ac.uk/cogent.php) in the MATLAB (Mathworks) environment.

### Procedure

In each scanning run, participants viewed one trial each of the four stimulus conditions (low and high spatial frequency through the left and right eye, presented in a pseudo-randomized order). Each individual trial lasted 19.2 s and trials were interleaved by 19.2 s blank periods during which only the fusion rings and the fixation cross were being presented. All participants were scanned on 12 runs of the experiment. During each block participants were required to press one of two keys on a MRI-compatible button box to indicate whether they thought the stimulus had been presented to their left or right eye.

In addition to the main experiment, to map the boundaries of the early retinotopic visual areas, in two additional runs we presented 19.2 s blocks of two large wedges of a contrast-reversing checkerboard pattern (8 Hz, subtending 2°–8° eccentricity) that alternated between the horizontal and vertical meridian of the visual field. All stimulus blocks were interleaved with 19.2 s blocks of fixation.

### Data acquisition

Blood Oxygen Level Dependent (BOLD) signals from visual cortex were measured using a 3T Allegra head scanner (Siemens Medical Systems, Erlangen, Germany), with a standard transmit-receive head coil and a single-shot gradient echo isotropic high-resolution EPI sequence (matrix size: 128×128; FOV: 192×192 mm^2^; in-plane resolution: 1.5×1.5 mm^2^; 32 oblique transverse slices with interleaved acquisition; slice thickness: 1.5 mm, no gap; TE: 30 ms; acquisition time per slice: 100 ms; TR: 3200 ms; echo spacing: 560 µs; receiver bandwidth: 250 kHz; 30% ramp sampling; 2-fold read oversampling to allow for k-space re-gridding; read gradient amplitude: 34.47 mT/m; read gradient slew rate: 344.7 mT/m/ms; flip angle α = 90°-). Slices were angled slightly to cover the calcarine sulcus. Real-time reconstruction was performed for quality assurance of the EPI data [19].

In each of the 12 scanning runs in the main experiment we acquired 58 volumes. In the retinotopic mapping experiments we acquired 124 volumes per run. The first four volumes (i.e. 12.8 s) were removed from any subsequent analysis to allow for T1 equilibration. To correct for EPI distortions induced by susceptibility artifacts, we acquired double echo FLASH images to estimate maps of the B_0_ field. Finally, we acquired T1-weighted anatomical images using a MDEFT sequence.

### Initial data analysis

Neuroimaging data were preprocessed and analyzed using SPM5 (http://www.fil.ion.ucl.ac.uk/spm). Functional images were corrected for slice acquisition time, realigned to the first image using an affine transformation to correct for small head movements and EPI distortions unwarped using B_0_ field maps. In order to reduce high-frequency noise, we applied moderate spatial smoothing with a kernel of 4 mm FWHM. Previous studies indicate that smoothing does not diminish decoding accuracies from visual cortex and can even be beneficial [20]–[22]. Consistent with this, we also obtained qualitatively similar results when using unsmoothed data.

The preprocessed images from the retinotopic mapping runs were entered into a general linear model specific to each participant with two regressors of interest corresponding to the vertical and horizontal stimulus conditions. Blocks were convolved with a canonical hemodynamic response function to generate regressors. From the anatomical images we reconstructed, inflated and flattened [23], [24] the grey-white matter boundary as a surface mesh for each cortical hemisphere using FreeSurfer (http://surfer.nmr.mgh.harvard.edu/fswiki). Linear contrasts between the two regressors were plotted on this surface. The boundaries of visual areas V1-3 were delineated by the activations corresponding to stimulation of the vertical and horizontal meridians. The surface vertices belonging to each visual area were projected back into volume space and the grey matter voxels falling in the space between these vertices on the grey-white matter surface and the pial surface were saved as binary masks.

### Multivoxel pattern analysis

Preprocessed functional data were further analyzed using custom software written in MATLAB. The time course from each run was z-score normalized. For each ROI the data of voxels in each volume (shifted by 1 volume  = 3.2 s to account for the lag of the hemodynamic response) were extracted and vectorized. Volumes from the same block were averaged so that there was only one voxel pattern (henceforth ‘sample’) for each block.

These data were then used for multivoxel pattern analysis using a leave-one-run-out cross-validation procedure, i.e. samples from all except one run were assigned to a training set and the remaining samples were used as a test set. For each condition we calculated the mean sample across all samples in the training set. These constitute the template patterns for each condition. To classify we then calculated a linear correlation between each sample in the test set and the templates from the training set (for the univariate decoding analysis, when there was only one variable in the sample, we calculated the difference between the test sample and the template, rather than the correlation). A test sample was then assigned to the condition which produced the greater correlation coefficient (or the smaller difference, in the case of only one voxel). Decoding performance for each cross-validation was estimated as the proportion of correct classifications, and the final decoding accuracy was calculated by averaging performances from all twelve cross-validations.

Since we used high-resolution fMRI each ROI contained hundreds to thousands of voxels. In order to reduce the dimensionality of the data set, we first calculated a T-statistic for comparing the two conditions of interest using *only the training data* set. We then ranked the voxels based on the difference calculated exclusively on the training data in descending order (ignoring the sign of the T-statistic). Only voxels up to a pre-determined cut-off number were then included in the analysis. For all the results reported here, this cut-off was 100 voxels. Essentially, this method selects the most discriminative voxels in the training data, and then tests the assumption that these same voxels also provide discriminative information about the stimulus in the *independent* test data. Moreover, estimating voxel biases with univariate difference statistics is a biologically plausible model of the response patterns that one would expect from the anisotropic functional architecture for ocular dominance and spatial frequency. However, other methods such as linear discriminant analysis, a linear support vector machine, or a non-linear k-nearest-neighbor classifier obtained similar results as the simple pattern-correlation classifier, which is consistent with recent reports comparing these different classification algorithms [25].

### Behavioral replication outside the scanner

In addition, participants also performed a behavioral task outside the scanner in a darkened room. The task here was the same as during scanning with the exception that individual stimulus presentations were now very short (350 ms) and we presented 624 trials. On each trial participants were required to press one of two keys on a button box to indicate whether they thought the stimulus had been presented to their left or right eye. Different conditions were presented in a semi-randomized order with the constraint that the same condition was never presented twice in a row and that within a series of 8 trials the same spatial frequency was never repeated twice in a row. A table-mounted divider made from black foam board was used to aid stereo-fusion.

### Stereovision and eye-dominance

In addition to the utrocular discrimination experiments, we tested whether all of our participants had normal depth perception by means of a simple random dot stereogram displayed in our psychophysics setup. Participants were instructed to report whether they could see a square plane with a binocular disparity of ∼2 arcmin. Further, to determine each participant's dominant eye, we carried out a hole-in-the-card test [26]. Participants fixated an object through a ∼3-by-3 cm hole in a sheet of paper, held at arms length from their face. Initially, the hole allowed both eyes to see this object. Subsequently, the participant was instructed to move the paper closer to the face, until only one eye to saw the object – the dominant eye. This test was repeated three times and always replicated the eye initially identified as dominant. In all but one participant the dominant eye was opposite to their handedness.

## Results

### Decoding stimulus eye-of-origin

We used fMRI and a simple pattern-correlation classifier to decode voxel response patterns in retinotopic visual cortex to left and right eye stimulation using low and high spatial frequency gratings, respectively. For each participant, we took the accuracy for decoding the stimulus eye-of-origin at a pre-determined cut-off number of 100 voxels per brain area. Although the choice of this cut-off is necessarily a priori and somewhat arbitrary, we informally noted that decoding accuracy remained very stable across different numbers of voxels.

**Figure 2:**
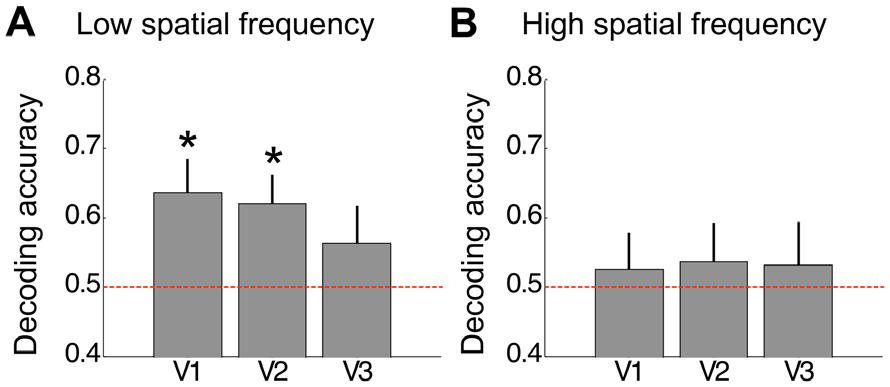
Decoding stimulus eye-of-origin for the low spatial frequency stimulus (A) and the high spatial frequency stimulus (B). Decoding accuracy (proportion correct) obtained for the 100 most discriminative voxels is plotted for the three ROIs averaged across participants. Error bars depict standard error of the mean. The asterisks indicate that accuracy was significantly above chance (permutation test, p<0.01, Bonferroni corrected).

Figure 2 plots the accuracy for decoding the stimulus eye-of-origin averaged across the group of participants for each of the retinotopic brain areas. Decoding was significantly (permutation test, p<0.01, Bonferroni corrected) better than chance for the low spatial frequency in V1 and V2 but not V3 (Figure 2A). On the other hand, when decoding the eye-of-origin of the high spatial frequency stimulus decoding accuracy was not above chance for any of the regions tested (Figure 2B). However, in a two-way repeated-measures ANOVA with factors spatial frequency and ROI there was no significant difference between the decoding accuracies obtained for the two spatial frequencies (F(1,7) = 1.51, p = 0.259) or between ROIs (F(2,14) = 0.23, p = 0.795) and no interaction of these terms (F(2,14) = 0.54, p = 0.595). This indicates that voxel patterns measured in response to either spatial frequency may contain a degree of discriminative information for the eye-of-origin. However, only for the low spatial frequency stimulus this was distinct enough to permit reliable decoding that was significantly better than chance.

**Figure 3:**
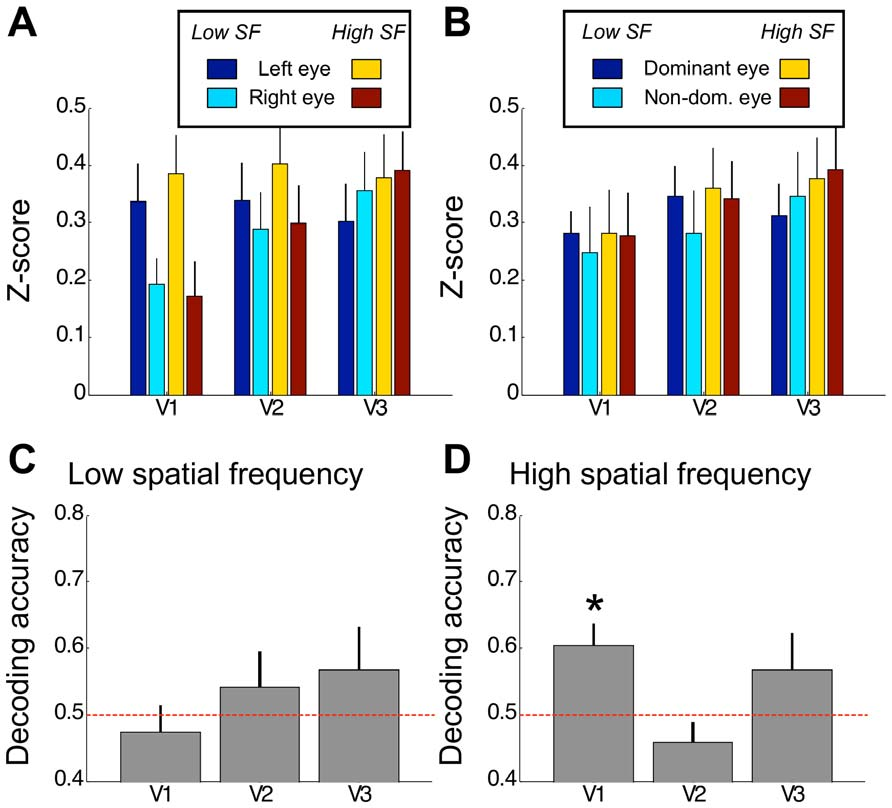
Mass-univariate response per condition. A–B. The signal change (z-score) averaged across the group of participants plotted for each ROI and stimulus condition. Data are grouped by anatomical eye (A) or by eye dominance (B). Error bars depict standard error of the mean. C–D. Univariate decoding stimulus eye-of-origin for the low spatial frequency stimulus (C) and the high spatial frequency stimulus (D). Decoding accuracy (proportion correct) obtained for the mean of the 100 most active voxels is plotted for the three ROIs averaged across participants. Error bars depict standard error of the mean. The asterisks indicate that accuracy was significantly above chance (permutation test, p<0.01, Bonferroni corrected).

The pattern-correlation algorithm we used for decoding implicitly normalizes the samples to the mean across voxels, which rules out that a simple difference in mean signal levels in a region of interest could account for our decoding results. However, this does not preclude the possibility that mean response levels could influence the gain of the pattern information in the visual cortex. If the overall response is weak the pattern of voxel biases may be obscured by measurement noise, but it may become more reliable with stronger responses – and thus easier to distinguish with our decoding algorithm. Therefore, in order to test whether our decoding results could be attributed to a difference in the mean signals evoked by our stimuli, we also analyzed the signal change (z-score) from the 100 most visually responsive voxels. In Figure 3A we plot the average signal across the group for each stimulus condition and each visual area examined. A three-way repeated-measures ANOVA with factors ROI, eye-of-origin, and spatial frequency showed that there was a difference in activation between ROIs (F(2,14) = 4.21, p = 0.037) but not between the two eyes (F(1,7) = 1.76, p = 0.226) nor between spatial frequencies (F(1,7) = 0.81, p = 0.399) showing that our small foveally presented stimulus evoked only a small change in mean BOLD signal that did not differ as a function of spatial frequency or eye-of-origin. There was, however, also a significant interaction between ROI and eye-of-origin (F(2,14) = 15.46, p<0.001), showing that there may been a small difference in the mean response to each eye in V1 compared to the other regions (Figure 3). However, critically, since there was no difference between spatial frequencies, but the MVPA showed reliable decoding of the eye-of-origin only for the low spatial frequency stimulus, this cannot explain our decoding results. Thus the ability to distinguish the eye-of-origin revealed by the MVPA arose from the local spatial pattern of activation that does not appear to depend on overall signal levels.

However, these results show that regardless of spatial frequency in V1, the mean response to the left eye was stronger than to the right eye (F(1,7) = 5.85, p = 0.046). Since the majority of our participants were left eye dominant we wondered if this difference in response strengths was due to eye dominance. We therefore reanalyzed the mean signals with respect to the dominant eye. Interestingly, this completely eliminated the differences we observed between eyes in V1 (Figure 3B). We only observed a significant difference between ROIs (F(2,14) = 4.21, p = 0.037), but not between the eyes (F(1,7) = 0.04, p = 0.857) or spatial frequencies (F(1,7) = 0.81, p = 0.399). Crucially, there now was no interaction between ROI and eye-of-origin (F(2,14) = 0.52, p = 0.605). This indicates that the differences in the fMRI response we measured between left and right eye stimulation do not relate to eye dominance.

These analyses of response strengths are necessarily at the group level. We also sought to perform an analysis of the mean response levels at the level of individual participants. We conducted a univariate decoding analysis for each participant: instead of decoding the stimulus eye-of-origin based on voxel *patterns*, we first averaged the responses across voxels in each sample before decoding. This analysis was consistent with the findings from the group analysis (Figure 3). We observed significant decoding of eye-of-origin from responses to high spatial frequency stimuli in V1 (permutation test, p<0.01, Bonferroni corrected), but not in any other region (Figure 3D). For the low spatial frequency stimuli decoding was not above chance levels (Figure 3C). However, there were no significant differences in accuracy between spatial frequencies (F(1,7) = 0.09, p = 0.767) or ROIs (F(2,14) = 2.66, p = 0.105), and no interaction (F(2,14) = 2.1, p = 0.159). We therefore cannot infer that decoding was better for high than low spatial frequencies. This however, is consistent with the results of the mean signal levels where we did not observe a difference between spatial frequencies but only between eyes.

### Utrocular discrimination

During the scan, as well as in an additional psychophysical test outside the scanner, participants performed a utrocular discrimination task. The stimuli outside the scanner were identical to those presented in the fMRI experiment, but shown for much shorter durations. Participants were required to indicate by means of a button press whether the grating had been presented to the left or the right eye.

**Figure 4:**
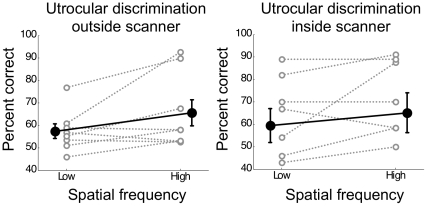
Utrocular discrimination outside the scanner and inside the scanner. Behavioral accuracy for judging which eye saw the grating stimulus is plotted for the low and high spatial frequency. Grey lines and open circles denote the performance for individual participants. Black line and solid circles denote the mean across participants (error bars depict standard error of the mean).

Performance on this task outside the scanner varied substantially between participants (Figure 4). Averaged across participants utrocular discrimination for the low spatial frequency was 57.3±3.2% (SEM) and for the high spatial frequency 65.5±5.9%. Performance was significantly greater than chance for the high spatial frequency (t(7) = 2.66, p = 0.033) and there was a trend towards significance for the low spatial frequency (t(7) = 2.26, p = 0.058). We only found a small difference in accuracy comparing high and low spatial frequencies with a trend towards statistical significance (t(7) = 2.04, p = 0.081) suggesting that performance for the high spatial frequency was slightly higher than for the low spatial frequency. In addition, we conducted sensitivity analysis on this behavioral data. We found that sensitivity, *d'*, for detecting stimulation of the dominant eye was −0.13±0.23 for low and 0.96±0.39 for high spatial frequency stimuli, respectively. Consistent with the raw accuracies, performance was therefore on average greater for high than low spatial frequency stimuli, but this difference only trended towards statistical significance (t(7) = −1.82, p = 0.111). Importantly, however, there was no significant difference in response bias between spatial frequencies (low: 0.07±0.13; high: 0.16±0.18; t(7) = −0.72, p = 0.496).

Estimating behavioral performance in the scanner was complicated by the small amount of data available. Each 19.2s stimulus block constituted one behavioral trial. Nonetheless, we found good agreement between our behavioral measurements outside the scanner and during the scan (Figure 4). Performance inside and outside the scanner was not significantly different (F(1,7)<1, p = 0.9). Further, there was a significant correlation between the two measures (R = 0.593, p = 0.016), which implies that our behavioral measurements outside the scanner were a reliable indicator of the utrocular discrimination performance during the scan.

### Decoding stimulus spatial frequency

**Figure 5:**
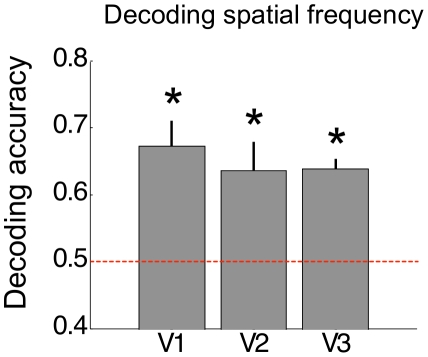
Decoding the spatial frequency. Accuracy obtained at 100 voxels (averaged across participants) for decoding between low and high spatial frequency is plotted for each ROI. Error bars depict standard error of the mean. Asterisks indicate whether accuracy was significantly above chance (permutation test, p<0.01, Bonferroni corrected).

Finally, for completeness we also determined whether it was possible to decode which spatial frequency was presented when pooling data from left and right eye stimulation across participants (Figure 5). We observed above chance decoding for all the retinotopic visual areas.

## Discussion

Here we employed fMRI and multivoxel pattern analysis in human participants to address whether decoding the eye-of-origin of a monocularly presented grating from BOLD signals in early visual areas depended on the spatial frequency of the stimulus. We found that in V1 and V2 decoding performance was significantly greater than chance levels for a low spatial frequency stimulus. This is consistent with previous studies suggesting that monocular neurons prefer low spatial frequencies [8], [9], [13]–[15]. Moreover, we found that the mean fMRI response in V1 differed between the two eyes. However, we did not observe significant differences in either the decoding performance or the overall signal levels between spatial frequencies.

Further, we tested whether participants could successfully discriminate the eye to which the stimulus had been presented. At the group level, utrocular discrimination was better than chance. Earlier studies reported that in normal participants utrocular discrimination is spatial frequency-dependent with good performance for low spatial frequency stimuli but chance performance for high spatial frequencies [5], [4]. We did not replicate these findings. If anything, we found that performance for the high spatial frequency stimuli was moderately better than that for the low spatial frequency, which is the opposite of those earlier findings. One possible reason for the differences between our findings and those in earlier studies may relate to differences in the stimuli employed in each study [5], [4]. Our low spatial frequency was at the low end of the range tested by Blake and Cormack (0.5 cycles/°-). Our high spatial frequency, however, was only 3.6 cycles/°-, because the projection system in the scanner environment placed an upper limit on the spatial frequency that could be used. At around 3.6 cycles/°- many of Blake and Cormack's participants showed above chance performance on utrocular discrimination, which could explain why our participants could judge the eye-of-origin at that spatial frequency. However, it is unlikely that this is the only explanation for the discrepancy between the studies, because we also did not replicate the very high accuracies for the low spatial frequency reported previously.

Our stimulus was also about half as wide as Blake and Cormack's with a standard deviation of 0.7°- of visual angle compared to 1.5°- [5], because this was the largest stimulus that allowed reliable stereo fusion and could be presented in the scanner setup. A larger stimulus produces a signal of greater spatial extent in retinotopic visual cortex which may aid utrocular discrimination. The fact that we held spatial phase of the Gabor stimuli constant probably resulted in adaptation, which may also have reduced signal strength somewhat. Furthermore, we used flickering gratings with a temporal frequency of 5 Hz. Utrocular discrimination for high spatial frequencies improves with increasing temporal frequency [5], although this again fails to explain why we did not find better behavioral performance for the low spatial frequency.

Finally, inside the scanner, our stimulus duration of 19.2 s was of course much longer than that used in previous studies. Utrocular discrimination performance saturates at stimulus durations of greater than 500 ms [5]. We found no differences in behavioral performance inside and outside the scanner, when stimulus duration was short (350 ms). Therefore, stimulus duration cannot account for differences between our behavioral findings and earlier studies. However, it is possible that the long stimulus exposure produced more robust eye specific signals in the visual cortex, which underlie our decoding results. In contrast, the behavioral decision of our participants as to which eye was being stimulated may be mediated by early transient signals that are blurred by the sluggish hemodynamic response.

Another important difference between the present study and those earlier studies [5], [4] is not directly related to the stimulus but to the experimental setup: we asked participants to view the monocular stimuli with free cross-fusion aided by a black foam board divider. It is possible that eye specific signals during free fusion are different than when viewing a dichoptic stimulus in a mirror stereoscope. The maintenance of free fusion requires the participant to make voluntary vergence eye movements. These may differ slightly between monocular stimulation of the left or right eye resulting in extra-retinal signals from the eye muscles that could be used for utrocular discrimination. It is conceivable, though perhaps less likely, that such signals are more pronounced for high spatial frequency stimuli. In any case, such an explanation is in line with the fact that we failed to find a relationship between decoding of the eye-of-origin in visual cortex and utrocular discrimination.

Importantly, there have also been behavioral studies challenging the idea that pattern-responsive neurons in the visual cortex are involved in judging the eye-of-origin [27], [28]. These experiments replicated the spatial frequency dependency of utrocular discrimination for simple grating stimuli reported by Blake and Cormack [4]. However, they suggested that awareness of the stimulated eye was due to differences in local luminance between the eyes [28]. They demonstrated that above chance utrocular discrimination for low spatial frequency gratings could be abolished by adding a luminance change to the other eye containing only a uniform grey. They interpreted this as evidence that the decision as to which eye is being stimulated was made solely based on a simple comparison of the light levels between the eyes. This, however, does not rule out a role of visual cortex in utrocular judgments. It merely suggests that it is mediated by simple luminance detectors, rather than by neurons tuned to specific spatial frequencies and orientations, which make up the majority of cells in the early visual cortex [11].

Our measurements of the overall fMRI signal in V1 are consistent with such an explanation. We found a significant difference in the responses to separate stimulation of the two eyes. Just as our participants' ability to judge the eye-of-origin, this difference did not depend on spatial frequency. If anything, utrocular discrimination and the difference between left and right eye responses were marginally (albeit non-significantly) greater for high spatial frequency. It is therefore possible that the overall signal in V1 is used for utrocular judgments. A difference in overall signal may result from differences in local luminance between the two eyes. However, it may also be indirectly related to extra-retinal factors such as differences in eye vergence, accommodation, or micro-saccades [28], [27].

Blake and Cormack [4] hypothesized that monocular neuronal populations underlie eye-of-origin judgments, because stereoblind participants showed superior utrocular discrimination compared to controls. Because the stereoblind visual system contains mostly monocular neurons [6], [7], they argued that these participants are able to access eye-of-origin information unavailable to normal participants. In stereoblind participants monocular stimulation is likely to produce even greater differences in the mean response of V1, which may provide a distinct signal that can be read out by decision making processes. In addition, a more sharply segregated ocular dominance map, due to the loss of binocular neurons, may also give rise to distinct pattern-information that could be exploited by the decoding analysis. However, our results indicate that in participants *with normal binocular vision*, the fine-grained pattern of monocular signals is probably not used for utrocular discrimination. This is also consistent with Blake and Cormack's finding that interocular transfer of visual aftereffects, regarded as a behavioral test of neuronal binocularity, is not dependent on spatial frequency [5].

### Decoding monocular signals in the visual system

A number of previous studies investigated eye specific signals in the human visual system with fMRI. In particular, one previous study employing multivoxel pattern analysis during binocular rivalry showed that it is possible to decode the currently perceived stimulus from response patterns in early visual cortex [29]. Their findings suggested the presence of eye specific responses in V1 (see Supplementary Data in that earlier publication), but because stimuli also differed in color and direction of rotation, it could not be entirely ruled out that successful decoding of rivalrous perception also relied partly on these attributes of the stimulus.

What underpins accurate decoding of the stimulated eye in the earlier regions? Ocular dominance columns in human V1 have a width of approximately 800 µm [14]. Using high-field fMRI and high-resolution EPI sequences it is possible to resolve individual ocular dominance columns [30], [31]. While our scanning setup at 3T did not permit the acquisition of such high-resolution images, we used an isotropic resolution of 1.5×1.5×1.5 mm^3^ with only moderate smoothing (4 mm FWHM kernel), which should in principle allow voxel biases from the two eyes to arise due to biased sampling from the underlying ocular dominance architecture.

However, recent reports proposed that monocular signals measured with fMRI at 3T are dominated by biases existing at a larger scale than the columnar functional architecture [32]. It is for instance possible that large draining blood vessels exhibit biases towards one or the other eye. One reason for the assertion of large scale structure is that even small head motion artifacts common in human fMRI studies would alter the biased sampling of the columnar structure by the coarse voxel grid [33]. The existence of more large scale biases is also supported by the findings that for decoding other stimulus dimensions, such as orientation and direction of motion [34], [20], [35], [21], [22], spatial smoothing does not diminish decoding accuracy and can even improve it. Importantly, the most discriminative voxels for stimulus orientation form elongated regions that correspond well to draining vessels [34]. In the context of decoding the eye-of-origin, such vascular biases may on the one hand arise from large-scale differences such as a contralateral bias of eye signals between the left and right hemisphere. However, since a particular vein may drain regions containing predominantly one type of column, the selectivity of blood vessels may be a functional marker of an anisotropic stimulus representation, such as the columnar architecture for ocular dominance in the primary visual cortex. Another possibility is that, independent of blood vessels, there also exist biases in the columnar structure at lower frequency harmonics and that these harmonics are sampled by the voxel grid [21]. Mathematical modeling suggested that for decoding of eye-of-origin from human visual cortex this is indeed the case: in the model, voxel biases arise due to local irregularities in the ocular dominance map resulting in low frequency components that can be detected with conventional voxel sizes at 3T [36].

Optical imaging and anatomical studies show that low spatial frequency domains fall at the center of ocular dominance columns meaning that neurons tuned to low spatial frequencies are predominantly monocular [8], [9], [15]. This is consistent with our observation in the present study that decoding of the eye-of-origin was successful only for the low spatial frequency stimulus. Even though we found no difference in decoding performance between spatial frequencies, we interpret this as an indication that voxel response patterns for monocular stimulation are indeed related to the ocular dominance map. First, even though neurons tuned to high spatial frequencies tend to be more binocular, this does not preclude weak ocular dominance biases. This may be in particular the case because our high spatial frequency (3.6 cycles/°-) was nowhere near the visual acuity limit. Second, the lack of a significant difference between spatial frequencies may also reflect a lack of statistical power. Whether the pattern-information detected by our decoding analysis is caused by biased sampling of individual columns or lower frequency harmonics of the columnar map, an indirect consequence of the draining veins supporting these columns, or if there is a more complex relationship between voxel responses and cortical architecture [33] is an interesting question to be answered by future research.

Finally, we also used MVPA to decode the spatial frequency of the stimulus when pooling data from left and right eye stimulation. For all the visual areas tested we found very robust decoding of spatial frequency. This is consistent with the presence of spatial frequency domains in these areas [15], [10] and with the fact that stimulus spatial frequency is mapped in early retinotopic cortex [37]. This result further replicates previous work from our laboratory [38] and confirms the reliability of our decoding method.

### Conclusions

Here we showed successful decoding of the eye-of-origin of a small grating stimulus from voxel patterns in human V1 for a low spatial frequency stimulus. However, we found no systematic relationship between decoding accuracy and the ability to judge which of the eyes saw the stimulus. Instead, we observed a difference in overall responses to stimulation of each eye, which is more in line with our behavioral results. We surmise that this signal is related to utrocular discrimination. Using an event-related design, future research should test whether this signal is indeed a neural correlate of utrocular judgments.
